# The BBaRTS Healthy Teeth Behaviour Change Programme for preventing dental caries in primary school children: study protocol for a cluster randomised controlled trial

**DOI:** 10.1186/s13063-016-1226-3

**Published:** 2016-02-20

**Authors:** Cynthia Pine, Pauline Adair, Louise Robinson, Girvan Burnside, Paula Moynihan, William Wade, James Kistler, Morag Curnow, Mary Henderson

**Affiliations:** Institute of Dentistry, Barts and The London School of Medicine and Dentistry, Queen Mary University of London, Whitechapel, London, E1 2BA United Kingdom; R&D Department, Salford Royal NHS Foundation Trust, Mayo Building, 3rd Floor, Stott Lane, Salford, M6 8HD United Kingdom; Health Psychology and Behavioural Medicine Research Group, School of Psychological Sciences and Health, University of Strathclyde, 40 George Street, Glasgow, G1 1QE United Kingdom; Department of Biostatistics, University of Liverpool, Institute of Translational Medicine, Crown Street, Liverpool, L69 3BX United Kingdom; Centre for Oral Health Research, Institute of Health and Society, Newcastle University, Framington Place, Newcastle upon Tyne, NE24BW United Kingdom; Centre for Immunobiology, Blizard Institute, Barts and The London School of Medicine and Dentistry, Queen Mary University of London, 4 Newark Street, London, E1 2AT United Kingdom; NHS Tayside, Tweed Place, Perth, PH1 1TJ United Kingdom; Kent Community Health NHS Foundation Trust, Capital House, Jubilee Way, Faversham, Kent, ME 13 8GD United Kingdom

**Keywords:** Dental caries, Behaviour change, Storybooks, Fluoride toothpaste, Free sugars, Microbiota

## Abstract

**Background:**

Oral health behaviours such as establishing twice-daily toothbrushing and sugar control intake need parental self-efficacy (PSE) to prevent the development of childhood dental caries. A previous study has shown that behaviour change techniques (BCTs) delivered via a storybook can improve parental self-efficacy to undertake twice-daily toothbrushing. Objective: to determine whether an intervention (BBaRTS, Bedtime Brush and Read Together to Sleep), designed to increase PSE; delivered through storybooks with embedded BCTs, parenting skills and oral health messages, can improve child oral health compared to (1) an exactly similar intervention containing no behaviour change techniques, and (2) the BBaRTS intervention supplemented with home supply of fluoride toothpaste and supervised toothbrushing on schooldays.

**Methods/Design:**

A 2-year, three-arm, multicentre, cluster randomised controlled trial. Participants: children (estimated 2000–2600) aged 5–7 years and their families from 60 UK primary schools. Intervention: Test group 1: a series of eight children’s storybooks developed by a psychologist, public health dentist, science educator, children’s author and illustrators, with guidance from the Department for Education (England). The books feature animal characters and contain embedded dental health messages, parenting skills and BCTs to promote good oral health routines focused on controlling sugar intake and toothbrushing, as well as reading at bedtime. Books are given out over 2 years. Test group 2: as Test group 1 plus home supplies of fluoride toothpaste (1000 ppmF), and daily supervised toothbrushing in school on schooldays. Active Control group: series of eight books with exactly the same stories, characters and illustrations, but without BCTs, dental health messages or parenting skills. Annual child dental examinations and parental questionnaires will be undertaken. A sub-set of participants will be invited to join an embedded study of the child’s diet and salivary microbiota composition. Primary outcome measure: dental caries experience in permanent teeth at age 7–8 years.

**Discussion:**

A multi-disciplinary team was established to develop the BBaRTS Children’s Healthy Teeth Programme. The books were developed in partnership with the Department for Education (England), informed by a series of focus groups with children, teachers and parents.

**Trial registration:**

ISRCTN21461006 (date of registration 23 September 2015).

## Background

Past caries experience in the primary teeth is the strongest predictor of caries occurrence in the permanent dentition [1]. The first permanent molar teeth, which are the most caries-prone teeth, begin to erupt after the fifth birthday and are mainly fully erupted during the sixth year [2]. They are most vulnerable to caries development within the first year of eruption [3]. Decay experience in these teeth account for the majority of decay in children and result in lifelong impacts and costs [4]. Therefore, preventing decay experience in these teeth would result in significant savings to dental service costs and enhanced children’s oral quality of life.

Dental caries is an entirely preventable disease and there is comprehensive guidance on prevention for dental teams working in the National Health Service (NHS) in England [5] and in Scotland [6] to advise families on brushing twice daily with fluoridated toothpaste, controlling dietary sugars’ intake especially at bedtime, and for those children at high risk, (e.g. with caries experience in primary teeth) to provide evidence-based clinical procedures including fissure sealants and fluoride varnish. National guidance has been developed for the frequency of dental attendance and recall linked to disease risk category [7] and a systematic use of risk categorisation is being piloted in the new dental contract in England [8, 9]. Dental caries preventive guidance is aligned with general public health recommendations for adopting a healthy diet low in free sugars to prevent obesity [10]. These general health messages and supporting skills are delivered in a wide range of both local authority and NHS programmes and settings.

However, there is strong evidence of stark oral health inequalities rooted in the social determinants of oral and general health. For caries development, the difference between a healthy state and disease initiation and progression relates to the balance between the amount and frequency of consumption of free sugars, and other potentially fermentable carbohydrates, that are metabolised by the bacteria in dental plaque producing acids as metabolic bi-products, leading to demineralisation of teeth and the presence of a favourable oral environment for remineralisation including adequate levels of fluoride [11]. In the absence of water fluoridation, the population approach to optimise fluoride exposure is through the establishment and maintenance of twice-daily toothbrushing with fluoridated toothpaste [12]. Both control of free sugars’ intake, especially at bedtime, and twice-daily brushing rely on the development and maintenance of healthy routines at home from a young age. The likelihood of these occurring is socially and culturally patterned, and depends on parental self-efficacy to establish the behaviours [13] with a supportive personal and community environment that sees these as normal and provides the skills and materials to make these choices natural and accessible.

However, in socially disadvantaged communities, there are significant barriers to establishing healthy behaviours and providing supportive community environments, leading to oral health inequalities, and unequal uptake of dental care. Bringing preventive methods into school settings can be an effective route to supplement and support behaviours and care, which may not have developed into consistent daily routines at home. Providing a home-to-school support programme can promote the development of oral health [14, 15]. The consequences of dental caries in schoolchildren manifests in pain and sepsis; lost days from school for children; and from work for parents and carers [16]. The effects of poor dental health in primary school children impact daily living and functioning; cause lost time and concentration on learning; increased costs to primary medical and dental care; and for those worst affected increased hospital costs due to dental extractions under general anaesthesia (GA) resulting in short-term and lifelong impacts [16–19]. Therefore, approaches which only treat the symptoms of the disease are costly both to individual children and families, and also to the NHS.

Different methods of primary and secondary prevention are advocated [11]. A medical model approach seeks to identify those at high risk of the disease and treat them, as with the approach to high serum cholesterol [20]. A population approach seeks to change the ambient environment both in terms of reducing the availability and acceptability of foods and drinks containing free sugars (e.g. by increasing their cost) and by enhancing community availability of fluoride (e.g. by increasing use of fluoride toothpaste through community programmes). The former, medical model approach would require a commissioner of NHS dental services to consider how dental contracts might be structured to enhance access to services and provide care. The latter, population approach, might engage local authorities (in England) in deciding on the type of community intervention to support to promote the oral health of schoolchildren. It is, therefore, critical to determine both the cost and effectiveness of different approaches, particularly in an environment of limited resource where there is a need to optimise investment. There is limited information available of the highest quality to accurately inform this choice for those commissioning services in these disadvantaged communities [21].

The design of this study has been based on the results of a major international study which indicated that the most significant variable predicting whether children were most likely to be caries-free was parents’ perception of their ability to successfully deliver child toothbrushing and control of sugar eating and drinking (‘sugar snacking’) behaviours, i.e. parental self-efficacy [22]. A previous proof-of-concept study by the authors has shown that a storybook approach can be used to improve parental self-efficacy to undertake twice-daily toothbrushing [23]. The age of the children at commencement of this trial has been chosen to be 5–7 years. Children begin school in the UK at age 5 years, and between 5 and 7 years their first permanent molar teeth erupt [24]. Therefore, an intervention study conducted to include this post-eruption period provides a pivotal time to evaluate primary prevention of dental caries in permanent teeth.

### Aim

The primary aim of this research is to determine whether children’s dental health can be improved by increasing parental self-efficacy using a storybook approach for two child behaviours: toothbrushing and reducing consumption of free sugars. Specifically, with the following objective:

### Objective

To determine whether an intervention (Test group 1) designed to increase parental self-efficacy using a storybook approach with embedded behaviour change techniques (BCTs) for two child behaviours (toothbrushing with fluoride toothpaste and reducing consumption of free sugars to within recommended levels [25], especially at bedtime) can improve child oral health compared to (1) an exactly similar intervention without embedded BCTs (Active Control) and, (2) the intervention (Test group 1) supplemented with home supply of fluoride toothpaste and supervised toothbrushing on schooldays (Test group 2).

### Research questions

- Can child dental health be improved by increasing parental self-efficacy using a behaviour change intervention delivered by storybooks for two child behaviours: toothbrushing and ‘sugar snacking’? (Control versus Test group 1)
- Can child dental health be improved by an enhanced intervention designed to improve parental self-efficacy using a behaviour change intervention delivered by storybooks for two child behaviours: toothbrushing and control of ‘sugar snacking’ *plus* home supply of fluoride toothpaste and supervised toothbrushing with fluoride toothpaste at school? (Control versus Test group 2)
- Does the addition of home supply of fluoride toothpaste and supervised toothbrushing with fluoride toothpaste at school increase the effectiveness of an intervention designed to improve parental self-efficacy using a storybook approach for two child behaviours: toothbrushing and ‘sugar snacking’? (Test group 1 versus Test group 2)

For a sub-study of the main study:

- Does an intervention designed to improve parental self-efficacy using a behaviour change intervention delivered by storybooks for two child behaviours: (toothbrushing and ‘sugar snacking’) result in changes to parental-reported child consumption of free sugars? (Control versus Test group 1)
- Does the addition of home supply of fluoride toothpaste and supervised toothbrushing with fluoride toothpaste at school increase the effectiveness of an intervention designed to improve parental self-efficacy using a storybook approach with embedded BCTs for two child behaviours (toothbrushing and ‘sugar snacking’) result in changes to parental-reported child consumption of free sugars? (Test group 1 versus Test group 2)
- Does an intervention designed to improve parental self-efficacy using a behaviour change intervention delivered by storybooks for two child behaviours (toothbrushing and ‘sugar snacking’) result in changes to the cariogenic profile of children’s microbiota? (Control versus Test group 1)
- Does the addition of home supply of fluoride toothpaste and supervised toothbrushing with fluoride toothpaste at school increase the effectiveness of an intervention designed to improve parental self-efficacy using a behaviour change intervention delivered by storybooks for two child behaviours (toothbrushing and ‘sugar snacking’) result in to the cariogenic profile of children’s microbiota? (Test group 1 versus Test group 2)

## Methods/Design

This is a three-arm, multicentre cluster randomised controlled trial (RCT), with blinded outcome assessment.

### Ethics, consent and permissions

Research ethics and governance approval has been obtained for this study from the Queen Mary Research Ethics Committee (ID: QMREC2013/43). Informed consent will be obtained for each participant.

### Main study sample and recruitment

Participants will be identified from primary schools in England and Scotland, whose head teachers have agreed to participate in the study. At the beginning of the school term parents of all children entering class 1 (5–6 years) will be sent an information pack and informed consent document via schools. All participants will be given reasonable time to consider the study and discuss with their family. Members of school staff will be able to answer questions and contact details for the study team will be on the information sheet.

Parents who complete the informed consent document and return it to the school will be given the baseline questionnaire to complete and return to the child’s teacher.

### Inclusion and exclusion criteria for main study

Inclusion criteria: children, aged 5–6 years, who are attending state-maintained primary schools in Kent and Newham in England, and Tayside in Scotland in which the school head teacher has given permission for their school to be included in the trial will be included in the study, subject to their parents giving written consent.

Exclusion criteria: children of the same age, in the same schools for whom parents have not given written permission for their children to take part.

### Sub-study sample, recruitment and data collection

Parents of participants in the schools in Tayside, Scotland who are already participating in The BBaRTS trial will be sent an information pack and informed consent document for the sub-study via schools. The sub-study will seek parental consent for collection of data on food and drink intake over a 3-day period using the Intake24 multiple-pass 24-hour recall system (https://intake24.co.uk) [26] or as a paper-based 3-day food and drink diary (to be entered into Intake24 by the researchers). Children will be included in the sub-study, subject to written parental consent; and will be excluded from joining the sub-study in the absence of written parental consent. In addition to parental completion of a 3-day food and drink diary, children included in the sub-study will also have a sample of unstimulated saliva collected at baseline and study end (after 2 years). It is expected that the Scottish cohort of the trial will include around 400 children and it is anticipated that about 50 % of their parents (*n* = 200) will give consent to join the sub-study.

The saliva samples will be collected using the following method. Sterile collection bottles will be prepared with a label affixed with the child’s identification (ID) number. The research dental nurse will have three items for each subject: a pre-labelled collection bottle, a sterile pipette and a small sterile sample bottle. The nurse will visit the schools and bring together, in small groups, the children from whom a saliva sample will be requested. Each child will be given a collection bottle and, under nurse supervision, the top of the bottle will be removed. Each child will be asked to lean their head forward and dribble saliva from their mouth into their collection bottle. No stimulation will be provided in the way of paraffin wax and children will be asked to gently drool the saliva naturally collecting in their mouth rather than spitting. Tissues will be provided to the children to wipe off excess saliva from their mouths. Once a sample exceeding 1 ml has been collected in the bottle, the research dental nurse will screw the top back onto each bottle. Any child who cannot provide a sample will be given extra time but if no sample is forthcoming, the child will be excused the collection and reassured. The children will be escorted back to their classroom and, before leaving the school, the nurse will pipette 1 ml of saliva from the collection bottle to the child’s small sample bottle, which the nurse will label with the child’s ID number. These small sample bottles will be put into a rack which allows the sample bottles to stand upright and be stored individually. The lid of the rack will be closed forming a container which will be put into a freezer box, which will contain frozen blocks. The freezer box will be transported back to the clinic base where the containers with the samples will be placed in a domestic refrigerator and transported under dry ice to the laboratory within 2 days. All samples will be placed in a freezer in the microbiology laboratory at −80 °C

### Data collection for main study

#### Primary outcome

Dental caries experience in permanent teeth at age 7–8 years.

Dental examinations will be conducted in the child’s school. The examinations will be conducted by independent dental examiners, trained in standardised dental epidemiological survey techniques [27] and blinded to the group allocation of the school. Dental caries experience on any surface in either dentition will be recorded. All children will be examined using sterilised or single-use mouth mirrors, Community Periodontal Index of Treatment Needs (CPITN) probes, a standardised dental examination lamp (2000 lux) and cotton wool rolls as needed.

This assessment will be analogous to national dental screening in schools, which is a simple visual assessment of the child’s teeth. Children will be asked to assent to the examination. Any child who does not assent and refuses to have their teeth checked will remain in the study and will not be excluded; the dental examination data will be recorded as missing data for that assessment. Teachers and parents will be informed prior to each assessment that these assessments are a simple observational screening and are not a replacement for the children’s dental examinations from their family dentist.

#### Secondary outcomes

Dental caries experience in permanent teeth at age 6–7 years (midpoint).

Oral cleanliness will be measured by plaque assessment on the buccal surfaces of upper anterior teeth at dental examinations [28].

Parents/guardians of children will be asked to complete a questionnaire pack at baseline, 1 year and 2 years post enrollment. This will be sent home and collected via the school. Questionnaires will take approximately 20 minutes to complete, incentives for completion of questionnaires will not be offered. Measures at 1 and 2 years will be secondary outcomes.

1. Oral Health Behaviours Questionnaire
  This is a validated measure examining parental attitudes, beliefs and behaviours towards dental care and includes parental self-efficacy [13]
2. Faces IV Family Satisfaction Scale
  This is a validated family self-report measure of family satisfaction with how the family operates on a day-to-day basis [29]
3. Early Childhood Oral Health Impact Scale
  This validated scale measures oral health-related quality of life [30]
4. Reading Intensity
  This scale is modified from earlier research undertaken by the team and measures parental- reported frequency of reading storybooks to their child [23]

#### Sub-study outcomes

1. Measurement of free sugars’ intake and other dietary variables.

Intake24 will be used to collect dietary information from a sub-sample of the study population. Intake 24 (https://intake24.co.uk) is a participant-completed computerised dietary recall system based on multiple-pass 24-hour recalls. In this study it will be used to collect dietary information over three consecutive days. From this, the energy intake (kcal/day) and the daily amount (g/day and percentage contribution to total energy intake) and daily frequency of intake of free sugars, the percentage contribution of specific food sources to free sugars’ intake will be derived. The intake of milk and intrinsic sugars (g/day and % energy), total sugars (g/day and % energy) intake of processed starchy foods (g/day and contribution to energy intake), intake of staple starch rich foods (staples) (g/day and % energy) will also be determined. The contribution to intakes made by defined food groups (e.g. biscuits and cakes, confectionery and drinks) will be determined.

2. Profile of oral microbiota, specifically *Streptococcus mutans,* lactobacilli and other caries-related bacterial taxa.

DNA will be extracted from unstimulated saliva samples and V1–V2 of the 16S ribosomal RNA genes of bacteria in the samples will be sequenced by means of the Illumina MiSeq platform using a dual-indexing barcoding approach. Sequencing will be performed using a 2 × 300 flow cell for paired-end sequencing. Sequences will be analysed using the mothur pipeline [31], following the MiSeq SOP. Operational taxonomic units (OTUs) will be constructed at the 98.5 % similarity level. OTUs will be identified with reference to the Human Oral Microbiome Database reference dataset [32, 33]. Distance matrices, generated with the Jaccard Index and thetaYC metric, will compare sample β-diversities based on OTUs and visualised as dendrograms and three-dimensional Principal Co-ordinates Analysis (PCoA) plots (generated in R). Analysis of Molecular Variance (AMOVA) will be performed to determine if clustering patterns seen in the PCoA plots are statistically supported by differences in the distance matrix. Differentially abundant OTUs between patient groups of interest will be identified using the LDA Effect Size (LEfSe) algorithm [34]. Proportions of mutans-group streptococci, lactobacilli, *Bifidobacteriaceae* and *Propionibacterium acidifaciens* will be compared across intervention groups using appropriate statistical tests depending on the data distribution.

### Randomisation

**Fig. 1:**
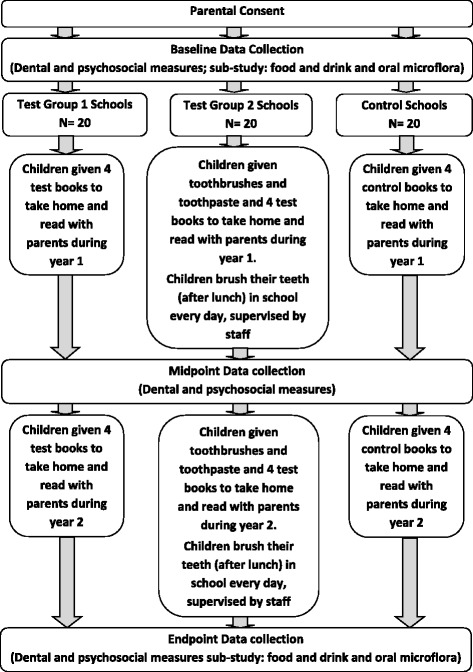
Bedtime Brush and Read Together to Sleep (BBaRTS) study flow diagram

Following the collection of consent forms from parents and collection of baseline dental and questionnaire data, schools will be randomised to Test group 1 (test books only), Test group 2 (test books and fluoride toothpaste), or Control (control books). The study flow in each group is presented in Fig. 1.

Randomisation will use block randomisation stratified by recruiting area, with variable block sizes.

### Interventions

#### Storybooks

A series of eight children’s books have been developed specifically for this study by a psychologist, public health dentist, science writer, children’s author, illustrators and with guidance from Department for Education (England) (DfE). These test books have the same animal characters in each story and contain dental health messages, parenting skills and BCTs embedded within to promote good oral health day and bedtime routines focused on controlling sugar intake and toothbrushing according to recommended levels [25] as well as reading (BBaRTS Programme: Bedtime: Brush and Read Together, Sleep). The books are designed to be read by parents to their children at bedtime. The books include behavioural and coping strategies, which are designed to target an increase in the parent’s confidence (self-efficacy) to adopt and routinise oral health behaviours for their child. Children in Test group 1 (test books) and Test group 2 (test books and fluoride toothpaste) will be given the eight books which will be distributed at 3-monthly intervals across 2 years of the study, i.e. one given to each child to take home each term of school.

Children in the control group will be given the series of eight *control* books, which have been developed to comprise exactly the same stories and illustrations but which exclude the specifics on oral health messages, parenting skills and BCTs. These storybooks will be given to the control group four times a year, in the same manner and frequency as the test books.

All parents will be sent a letter with each book, explaining that books are intended to be read to their child before bedtime.

#### Fluoride toothpaste and toothbrushing

Daily toothbrushing will be carried out at school for children in Test group 2 (books and fluoride toothpaste). The school will be provided with age-appropriate toothbrushes and fluoridated toothpaste (in line with Delivering Better Oral Health (DBOH) guidelines [5]). A brushing supervisor will be trained in school brushing methods in each school, including equipment storage and Case Report Form (CRF) completion. Children will also be provided with toothbrushes and fluoride toothpaste to take home every 3 months whilst taking part in the trial. Supplementary toothbrushing charts will be provided to support the home brushing component.

#### Sample size

Previous study and pilot study data on similar populations have shown that there is likely to be virtually no caries in first permanent molars at baseline, and most teeth will be unerupted [15]. As the study is cluster randomised, the intra-cluster correlation coefficient (ICC) must be considered when calculating sample size. Few estimates of school-level ICC are available for dental data, so a value of 0.01 was used, based on unpublished data collected by the authors. To allow for participant dropout and non-consent, it is assumed that an average of 30 pupils per school will participate and have primary outcome data available at study end. If the caries incidence in the control group is 35 %, the observed value in a previous study in a deprived population, a sample size of 19 schools per group allows detection of a reduction in caries to 25 % incidence in the test groups, with 80 % power, overall *α* = 0.05 (adjusted for multiple comparisons), and ICC = 0.01. For caries incidence in the control group of 20 % (the approximate value observed in the pilot study); the same sample size allows detection of a reduction to 12 % in the test groups. To allow for potential withdrawal of schools, one additional school will be recruited in each group; therefore, 60 primary schools will be randomly allocated to three clusters, taking area into account. If the assumption of 30 pupils per school holds, this will give a total sample size of 1800.

#### Method of analysis

A detailed statistical analysis plan will be prepared prior to data analysis. Comparison of the primary outcome variable between the groups will be conducted using multilevel logistic regression analysis, to produce estimates of odds ratios and confidence intervals, adjusting for baseline characteristics deemed to be potential confounding variables, and allowing for clustering effect. The closed testing principle will be used to ensure a familywise error rate of less than 5 %. If the overall null hypothesis of equality of all three groups is rejected at *α* = 0.05, then pairwise comparisons between groups will be carried out at *α* = 0.05. If the overall null hypothesis cannot be rejected, no pairwise comparisons will be carried out. The potential confounders will be specified prior to analysis, and may include deprivation, age, sex and eruption status. Comparisons of secondary outcome variables between groups will be carried out using multilevel logistic or linear regression, as appropriate. Sensitivity analyses of the possible effect of missing data will be carried out using multiple imputation techniques. Reporting of the trial will follow the Consolidated Standards of Reporting Trials (CONSORT) guidelines extension to cluster trials [35].

Independent analysis will be undertaken by psychologists not involved in the development of the books. A Delphi method will be used to validate the behaviour change techniques delivered by the intervention [36, 37].

## Discussion

### The process for development of the intervention

A multi-disciplinary team was established at Queen Mary University of London, Barts and the London Institute of Dentistry to develop the BBaRTS (Bedtime Brush and Read Together to Sleep) Children’s Healthy Teeth Programme. Continuing on from previous work [23] the team wanted to develop attractive, colourful, age-appropriate storybooks as the platform for delivering behaviour change to parents to enhance their self-efficacy in relation to their child’s twice-daily toothbrushing and control of ‘sugar snacking’. A review of children’s storybooks in relation to oral health has been undertaken and most focused on visits to the dentist with little to no reference to preventive behaviours [23]. Therefore, it was decided the focus of the books should incorporate preventive oral health behaviours and developmental changes (e.g. toothbrushing with fluoride toothpaste, controlling sugars in children’s foods and drinks; and losing primary teeth and growing new permanent teeth). Within existing children’s stories sustained situational interest can result in children developing a greater interest [38]; therefore, it was decided that a series of storybooks, revisiting the same characters would sustain the greatest level of interest. Previous research suggests that 6- to 8-year-olds prefer cartoons [39], so the style used was that of cartoon characters with vibrant colour schemes to attract young readers. It is noted that the books are designed to be read by parents to their child.

Initially two stories were written (Miles Salter) and illustrated (Pony Ltd.) in line with the brief above. Storybook 1 depicted anthropomorphised animal characters (frogs), Storybook 2 used illustrations of children dressed as animals (elephants). Both stories had easy-to-read, culture-neutral named characters, depicting toothbrushing behaviour and healthy food choices as a normal part of an exciting adventure story. To evaluate the appeal and acceptability of the storybooks, the research team organised focus groups in two London schools, with teachers and parents as well as children in class 1 (aged 5–6) and class 2 (aged 6–7), all of whom evaluated them.

Following analysis of focus group results and feedback it was decided that Storybook 1 should be taken forward as a concept for testing. In collaboration with literacy advisors at the DfE, the first two storybooks of the series of eight were written by Miles Salter (published author of children’s books), Sai Pathmanathan (science educator), Cynthia Pine (Professor in Dental Public Health) and Pauline Adair (clinical and health psychologist). Illustrations for the book were developed with local illustrators (Pony Ltd.) (http://www.ponybox.co.uk/), experienced in children’s education and research materials.

Literacy advisors at the DfE supported the development of the books and provided advice about the phonics, rhyme, rhythm and repetition and framing of the stories around events in children’s lives. They advised that the books were not suitable for children to read alone, due to the reading level, but supported the books as those to be taken home and read by parents to their child at bedtime. This aligns with existing literacy schemes in schools by encouraging children to read with parents at home. DfE colleagues recommended questions and information to guide the adult readers. These were subsequently added to the back of the book. Advice was also taken on the age-appropriateness of the book content and how this should develop over the series of eight books as the children get older and their cognitive development changes.

A proof-of-concept study was conducted with the first storybook in four primary schools in London UK, with a range of socioeconomic profiles. Schools were selected by the DfE and consultants in dental public health colleagues. The schools were all in inner London with a very considerable range of diversity and significant numbers of children and families whose first language was not English.

On completion of consent forms children (*n* = 144) were given a unique ID number, which was used for the duration of the study. Parents were given baseline questionnaires, adapted from existing oral health behaviours questionnaire [13] and reading scales [23] to complete and return to their child’s teacher. On return of the questionnaire each child was randomised to group 1 (Test) or group 2 (Control). Children in group 1 were provided with a copy of ‘*Boom and Bang with Zip and Pop*’ [40]. Children in group 2 (Control) were given a copy of ‘*Giraffes Can’t Danc*e’ [41], a popular children’s storybook containing no dental health messages, but a positive story containing animal characters involved in dance. After 1 month all books were collected and an endpoint questionnaire was handed out to all parents of children enrolled in the study. Additionally, five focus groups were conducted in two of the schools to explore the children’s (*n* = 28) opinions on books 1 and 2 of the ‘*Zip and Pop*’ series and the outlines for the remaining books in the series.

In all, 105 families completed the study, 54 in the test group and 51 in the control group. Seventy-one (68 %) of the families were of non-UK origin, 34 (63 %) in the test group, and 37 (73 %) in the control group. On a five-point scale (1 = strongly disagree; 5 = strongly agree), most families found the stories in both books were well-understood (mean (SD) 4.30 (0.64) Test; 4.34 (0.49) Control). When asked if they agreed the story helped them talk about brushing their child’s teeth: UK-origin parents had mean response of 3.55 (0.60) Test compared to 2.29 (1.07) Control; with 3.41 (0.99) Test and 3.25 (1.08) Control for non-UK- origin parents. The mean response to whether the story helped UK-origin parents to talk to their child about healthy foods and drinks was 3.70 (0.47) Test compared to 2.07 (1.14) Control; but 3.69 (0.93) Test and 3.50 (0.96) Control for non-UK-origin parents. UK-origin parents’ responses to whether reading the storybook together has helped their child co-operate better with getting their teeth brushed were 3.10 (0.64) Test versus 2.14 (0.86) Control; but non-UK-origin parents were 3.42 (0.97) Test and 3.27 (0.84) Control.

The books were both well-accepted and the children were very reluctant to return the test books at the end of the study. Parents accepted the study principles, were willing to be randomised to groups and welcomed the opportunity of receiving free storybooks. The community engagement to the feasibility study was deliberately challenging in terms of testing feasibility of an intervention set in English. Discussion with teachers and assistants identified that the schools had chosen to send home all school material in English as a mechanism to promote engagement with language development. The schools had developed extensive systems to support English language development. It is evident from the results of this study that taking the books into a substantive trial is feasible, that although there are challenges in using this medium for behaviour change for non-UK-origin families, the support systems within schools provide a mechanism to enhance engagement with reading.

### Trial status

This trial is currently recruiting participants.

## Abbreviations

BBaRTS: Bedtime, Brush and Read Together to Sleep
CRF: Case Report Form
DfE: Department for Education (England)

## References

[1] Li Y, Wang W (2002). Predicting caries in permanent teeth from caries in primary teeth: an eight-year cohort study. J Dent Res.

[2] Batchelor P, Sheiham A (2004). Grouping of tooth surfaces by susceptibility to caries: a study in 5–16 year-old children. BMC Oral Health.

[3] Härkänen T, Larmas MA, Virtanen JI, Arjas E (2002). Applying modern survival analysis methods to longitudinal dental caries studies. J Dent Res.

[4] Office for National Statistics. Children’s dental health in the United Kingdom, 2003. In: London: National Statistics; 2005

[5] Department of Health, British Association for the Study of Community Dentistry: delivering better oral health: an evidence-based toolkit for prevention. In: 2nd ed. 2009.

[6] Scottish Intercollegiate Guidelines Network (SIGN). Dental interventions to prevent caries in children. In: Edited by SIGN, vol. March 2014. Edinburgh; 2014

[7] National Institute for Clinical Excellence. Dental recall. Recall interval between routine dental examinations. In: London: NICE; 2004.

[8] Department of Health. NHS dental contract: proposals for pilots. In: 2010

[9] Department of Health. Dental quality and outcomes framework. In: 2010.

[10] Moynihan P, Kelly S (2013). Effect on caries of restricting sugars intake systematic review to inform WHO guidelines. J Dent Res.

[11] Featherstone JDB (2000). The science and practice of caries prevention. J. Am. Dent. Assoc..

[12] Burt B, Stephen E, Pine C, Harris R (2007). Community-based strategies for preventing dental caries. Community oral health.

[13] Adair PM, Pine CM, Burnside G, Nicoll AD, Gillett A, Anwar S (2004). Familial and cultural perceptions and beliefs of oral hygiene and dietary practices among ethnically and socio-economically diverse groups. Community Dent Health.

[14] Lister-Sharp D, Chapman S, Stewart-Brown S, Sowden A (1999). Health promoting schools and health promotion in schools: two systematic reviews. Health Technol Assess.

[15] Curnow MM, Pine CM, Burnside G, Nicholson JA, Chesters RK, Huntington E (2002). A randomised controlled trial of the efficacy of supervised toothbrushing in high-caries-risk children. Caries Res.

[16] Yusuf H, Gherunpong S, Sheiham A, Tsakos G (2006). Validation of an English version of the Child-OIDP index, an oral health-related quality of life measure for children. Health Qual Life Outcomes.

[17] Jameson K, Averley PA, Shackley P, Steele J (2007). A comparison of the ‘cost per child treated’ at a primary care-based sedation referral service, compared to a general anaesthetic in hospital. Br Dent J.

[18] Shepherd MA, Nadanovsky P, Sheiham A (1999). The prevalence and impact of dental pain in 8-year-old school children in Harrow. England British Dental Journal.

[19] RCS. The state of children’s oral health in England. In: Royal College of Surgeons. London, England: Faculty of Dental Surgery; 2015.

[20] Rose GA, Khaw K-T, Marmot MG (2008). Rose’s strategy of preventive medicine: the complete original text.

[21] Cooper AM, O’Malley LA, Elison SN, Armstrong R, Featherstone VA, Burnside G (2011). Primary school-based behavioural interventions for preventing caries. Cochrane Database of Systematic Reviews.

[22] Pine CM, Adair PM, Nicoll AD, Burnside G, Petersen PE, Beighton D (2004). International comparisons of health inequalities in childhood dental caries. Community Dent Health.

[23] O’Malley L (2013). The development and evaluation of a novel health promotion intervention (Kitten’s First Tooth) to improve children’s oral health in a deprived area of North West England.

[24] Ekstrand KR, Christiansen J, Christiansen MEC (2003). Time and duration of eruption of first and second permanent molars: a longitudinal investigation. Community Dent Oral Epidemiol.

[25] Department of Health. Delivering better oral health: an evidence based toolkit for prevention – 2nd ed. In: 2009.

[26] Mauerhoefer LKP, Poliakov I, Olivier P, Foster E (2014). Testing the feasibility of Intake24 as a portion size estimation aid. School of computing science technical report series.

[27] Pine C, Pitts N, Nugent Z (1997). British Association for the Study of Community Dentistry (BASCD) guidance on sampling for surveys of child dental health. A BASCD coordinated dental epidemiology programme quality standard. Community Dent Health.

[28] Silness J, Löe H (1964). Periodontal disease in pregnancy II. Correlation between oral hygiene and periodontal condition. Acta Odontol Scand.

[29] Olson CBS, V A, Merrill LL (2004). The influence of survey confidentiality and construct measurement in estimating rates of childhood victimization among Navy recruits. Military Psychology.

[30] Pahel BT, Rozier RG, Slade GD (2007). Parental perceptions of children's oral health: the Early Childhood Oral Health Impact Scale (ECOHIS). Health Qual Life Outcomes.

[31] Schloss PD, Westcott SL, Ryabin T, Hall JR, Hartmann M, Hollister EB (2009). Introducing mothur: open-source, platform-independent, community-supported software for describing and comparing microbial communities. Appl Environ Microbiol.

[32] Chen T, Yu WH, Izard J, Baranova OV, Lakshmanan A, Dewhirst FE (2010). The human oral microbiome database: a web accessible resource for investigating oral microbe taxonomic and genomic information. Database.

[33] Dewhirst FE, Chen T, Izard J, Paster BJ, Tanner AC, Yu WH (2010). The human oral microbiome. J Bacteriol.

[34] Segata N, Izard J, Waldron L, Gevers D, Miropolsky L, Garrett WS (2011). Metagenomic biomarker discovery and explanation. Genome Biol.

[35] Schulz KF, Altman DG, Moher D (2010). CONSORT 2010 statement: updated guidelines for reporting parallel group randomised trials. BMC Med.

[36] Hsu C-C, Sandford BA (2007). The Delphi technique: making sense of consensus. Practical Assessment, Research & Evaluation.

[37] Michie S, Ashford S, Sniehotta FF, Dombrowski SU, Bishop A, French DP (2011). A refined taxonomy of behaviour change techniques to help people change their physical activity and healthy eating behaviours: the CALO-RE taxonomy. Psychol Health.

[38] Grant L. Comparative evaluation of science communication activities and their impacts. Liverpool, England: University of Liverpool; 2005.

[39] Hodge RIV, Tripp D (1986). Children and television: a semiotic approach.

[40] Salter M, Pine C, Adair P, Pathmanathan S, Sweeney N, Boutrou O. Boom and Bang with Zip & Pop. London: Queen Mary University of London; 2013.

[41] Andreae G, Parker-Rees G. Giraffes Can't Dance. London: Orchard Books; 2014.

